# Plant beneficial microbiome a boon for improving multiple stress tolerance in plants

**DOI:** 10.3389/fpls.2023.1266182

**Published:** 2023-09-11

**Authors:** Sajad Ali, Anshika Tyagi, Rakeeb Ahmad Mir, Irfan A. Rather, Yasir Anwar, Henda Mahmoudi

**Affiliations:** ^1^ Department of Biotechnology, Yeungnam University, Gyeongsan, Gyeongbuk, Republic of Korea; ^2^ Department of Biotechnology, Central University of Kashmir, Ganderbal, India; ^3^ Department of Biological Sciences, Faculty of Science, King Abdulaziz University (KAU), Jeddah, Saudi Arabia; ^4^ Directorate of Programs, International Center for Biosaline Agriculture, Dubai, United Arab Emirates

**Keywords:** beneficial microbes, abiotic stressors, combined stress, climate change, tailored microbiota

## Abstract

Beneficial microbes or their products have been key drivers for improving adaptive and growth features in plants under biotic and abiotic stress conditions. However, the majority of these studies so far have been utilized against individual stressors. In comparison to individual stressors, the combination of many environmental stresses that plants experience has a greater detrimental effect on them and poses a threat to their existence. Therefore, there is a need to explore the beneficial microbiota against combined stressors or multiple stressors, as this will offer new possibilities for improving plant growth and multiple adaptive traits. However, recognition of the multifaceted core beneficial microbiota from plant microbiome under stress combinations will require a thorough understanding of the functional and mechanistic facets of plant microbiome interactions under different environmental conditions in addition to agronomic management practices. Also, the development of tailored beneficial multiple stress tolerant microbiota in sustainable agriculture necessitates new model systems and prioritizes agricultural microbiome research. In this review, we provided an update on the effect of combined stressors on plants and their microbiome structure. Next, we discussed the role of beneficial microbes in plant growth promotion and stress adaptation. We also discussed how plant-beneficial microbes can be utilized for mitigating multiple stresses in plants. Finally, we have highlighted some key points that warrant future investigation for exploring plant microbiome interactions under multiple stressors.

## Introduction

Plants are very often confronted by different biotic and abiotic stressors that can occur individually or in combination, impeding their growth and output (Eckardt et al., 2023; Tyagi et al., 2023). Global climate change has significantly increased the prevalence of multiple stressors in plants, which has a negative impact on agricultural growth and productivity (Eckardt et al., 2023). The unprecedented droughts, heat waves, fires, and floods across the world are some of the major consequences of global warming that have threatened agricultural productivity and the earth’s biodiversity (Zandalinas et al., 2021). Abiotic stressors such as drought, salinity, heat, cold, waterlogging, and heavy metals are known to cause 51–82% production losses thereby posing a serious threat to global food security for growing human populations (Oshunsanya et al., 2019). During their lifespan, plants are frequently subjected to recurrent combinations of abiotic stressors that occur simultaneously or in sequence. For instance, the occurrence of heat and drought stress is very often confronted by plants which lead to severe crop damage and yield losses (Mittler et al., 2012; Li et al., 2013; Sánchez-Bermúdez et al., 2022). According to Ahmed et al. (2013) the combination of drought and salinity stress causes more harm to barley plants than stress alone, mostly via impairing physiological and biochemical properties. Similarly in cotton plants, it was found that a combination of drought and heat stress causes more detrimental effects than individual stress by altering not only photosynthesis and stomatal activities but also increasing leaf temperature (Carmo-Silva et al., 2012; Melandri et al., 2021). Numerous studies have suggested that an increase in ambient temperature over time can endanger plant viability by increasing the frequency of droughts as well as the harmful effects of heat stress on plants (Mittler et al., 2012; Li et al., 2013; Matiu et al., 2017; Aubert and Quinet, 2022). According to Su et al. (2015), a combination of drought and cold stressors has a more detrimental impact on grape development and yield than individual stress. Another recognized effect of abiotic stress combination is ozone and cold stress on wheat plants which impairs their ability to withstand frost (Barnes et al., 1988). It was reported that salinity and ozone stress significantly lower the yields of *Oryza sativa* and *Cicer arietinum* (Welfare et al., 2002). Further, we have summarized the impact of combined abiotic stressors on different crops in **Table 1**. As it is difficult to predict how crops will respond to too many stress combinations and the ensuing damage from them, therefore, it is crucial to comprehend the intricate interactions between plants and multiple abiotic stress combinations (Anderegg et al., 2015). This is especially important given the urgency of global climate change that could make relationships more complicated by raising both the severity and frequency of adverse weather occurrences (Ju-Qi et al., 2022).

**Table 1:** Effect of combined abiotic stressors on plant morphological, physiological and biochemical traits.

Plant	Stress combinations	Impact on plant traits	References
Barley	Drought and salinity	Reduce chlorophyll content, inhibit plant growth, reduce net photosynthetic rate, osmotic potential and water potential	Ahmed et al., 2013
Arabidopsis	Drought + Heat	Inhibit photosynthesis, Decrease stomatal conductance Reduce spikelet fertility, grain numbers and overall yield	Prasad et al., 2011; Suzuki et al., 2014
Wheat	Heat and drought	Inhibit seedling growth	Keleş and Öncel, 2002
Rice	Drought + Heat	Decrease photosynthetic rate, reduced stomatal conductance and biomass	Dugasa et al., 2019; Da Costa et al., 2021
Canola	Drought + Heat	Inhibit root morphological and biochemical traits	Wu et al., 2017
Alpine	Drought + Heat	Affect photosynthesis activity	Ma et al., 2020
Sugarcane	Drought + Cold	Inhibit photosynthesis, impairments in CO_2_ assimilation	Sales et al., 2013
Chickpea	Drought + Cold	Alter physiological and biochemical traits	Kaur et al., 2016
Tomato	Heat +salt	Inhibit the activity of photosystem II Decrease CO_2_ assimilation	Rivero et al., 2014
Mungbean	Drought + Nutrient deficiency	Reduced rate of photosynthesis, transpiration, stomatal conductance, water use efficiency	Meena et al., 2021
Wheat	Drought and salinity	Inhibits plant growth, decreases photosynthetic and biomass	Dugasa et al., 2019
Barilla	Drought and salinity	Reduced root length, shoot height and biomass, decreased photosynthetic pigments	Lu et al., 2021
Pistachio	Salinity and drought	Alters root morphological and biochemical traits	Goharrizi et al., 2020
Triticale	salinity and drought	Reduced chlorophyll pigments, dry mass, RWC	Mohammadi Alagoz et al., 2023
Pea	Heavy metal+ UV	Decreased chlorophyll content, carbon fixation, reduced photosynthetic activity (PSI and PSII)	Srivastava et al., 2012
Maize	Drought and heat	Reduced root number	Vescio et al., 2021
Summer squash	Waterlogging and salinity	Inhibit root and shoot growth	Huang et al., 1995
Barley	Waterlogging and salinity	Reduced chlorophyll content, water content and plant biomass, retarded growth	Zeng et al., 2013
Tomato	Waterlogging and salinity	Decreased photosynthesis inhibit growth	Zhou et al., 2022

The occurrence of numerous stress combinations, such as biotic and abiotic stressors (pathogens, drought, salinity, and heat), or abiotic stressors (drought, salinity, and heat), could further reduce agricultural productivity. For instance, it has been shown that drought significantly worsens the damage caused by pathogens to aerial (leaf and stem) and underground (root) structures, ultimately leading to a higher plant mortality rate (Jactel et al., 2012). The incidence and severity of necrotrophic pathogens increased dramatically under drought conditions (Barradas et al., 2018). Similarly, salt stress in tomato plants increases the occurrence of Fusarium wilt fungal disease which leads to more detrimental impact on them (Daami-Remadi et al., 2009). Increasing temperature also leads plants more susceptible to diseases and also to the development of more virulent pathovars (Ladányi and Horváth, 2010). Global warming will also change the pathogen shift from cold to warm or vice versa which may pose a serious threat to agriculturally important crops (Burgess et al., 2017). Many reports have found that heat stress decreases the host’s immunity against different pathogens resulting in severe yield losses (Jactel et al., 2012). In the future unprecedented abiotic stressors will make plants more susceptible to diseases by promoting their reproduction, spread, and evolution into more dangerous pathogens (Ali et al., 2017; Matsuhashi et al., 2020).

Our understanding of plants’ response to individual stressors is well understood but we are for a way to understand how the plant responds to combined stressors. Given the multiple external pressures plants face pose not only a serious threat to their survival but also to food security and the agricultural economy. Hence it is important to decipher how plants respond to multiple stressors and also develop new tools that will provide multiple stress tolerance in plants. In the past, a number of tools were used to boost crop yield and stress tolerance in various crop systems, but all had drawbacks. For instance, the use of chemical fertilizers is currently an essential part of agriculture, but they ultimately cause soil pollution, degradation, nutrient pollution, eutrophication, and greenhouse gas emissions (Arif et al., 2020). Additionally, only 0.1% of chemical pesticides reach their intended target; the remainder seeps into the soil and water thereby damaging the ecosystem (Fukamachi, 2020). Although genetically engineered (GM) crops continue to be a viable choice as a low-input, sustainable agriculture technique, the research and regulation required to generate new cultivars are time-consuming and expensive and can take decades to achieve market and regulatory clearance (Ab Rahman et al., 2018). One way to meet these challenges is to harness and integrate beneficial microbiomes i.e., those that promote plant growth, nutrient uptake, soil fertility, and host resistance to biotic and abiotic stressors (Busby et al., 2017; Ali et al., 2022; Ali et al., 2023). In the natural world, plants respond and adapt to abiotic and biotic challenges in the presence of microbial communities that live inside and outside of the host plants. Therefore, it is important to comprehend how plants undergo microbiome reprogramming amid combined abiotic stressors and how they enriched distinctive microbial communities that will help them to cope with stress. Indeed, in the last two decades, there have been significant reports on exploring plant microbiome diversity and their functions under different growth and environmental conditions in both crop and model plants viz*.*, rice (Knief et al., 2012; Edwards et al., 2015); barley (Bulgarelli et al., 2015); corn (Aira et al., 2010); soybean (Rascovan et al., 2016); wheat (Donn et al., 2015); *Arabidopsis thaliana* (Bulgarelli et al., 2012). However, their expansion to the field has been limited when compared to classical PGPR microbes. There is still a long way to go before this knowledge can be effectively used in agricultural fields or in the development of tailored microbial consortia. Also, the successful incorporation of beneficial microbiomes into plant breeding and agronomical management practices is practically hampered due to the limited knowledge of plant microbiome interactions under different environmental stressors, host-microbe interactions, plant genotype variability, and microbe-microbe interactions in addition to lack of coordination between the academic scientific community and industrial researches. In this mini-review, we highlight the impact of combined stressors on sustainable agriculture and also highlight the importance of beneficial microbiota for combating multiple stressors. In addition, this review provides possible directions for future research for the development of tailored microbiota for tackling multiple stressors in sustainable agriculture.

## Revisiting the role of beneficial microbes in plants

**Figure 1:**
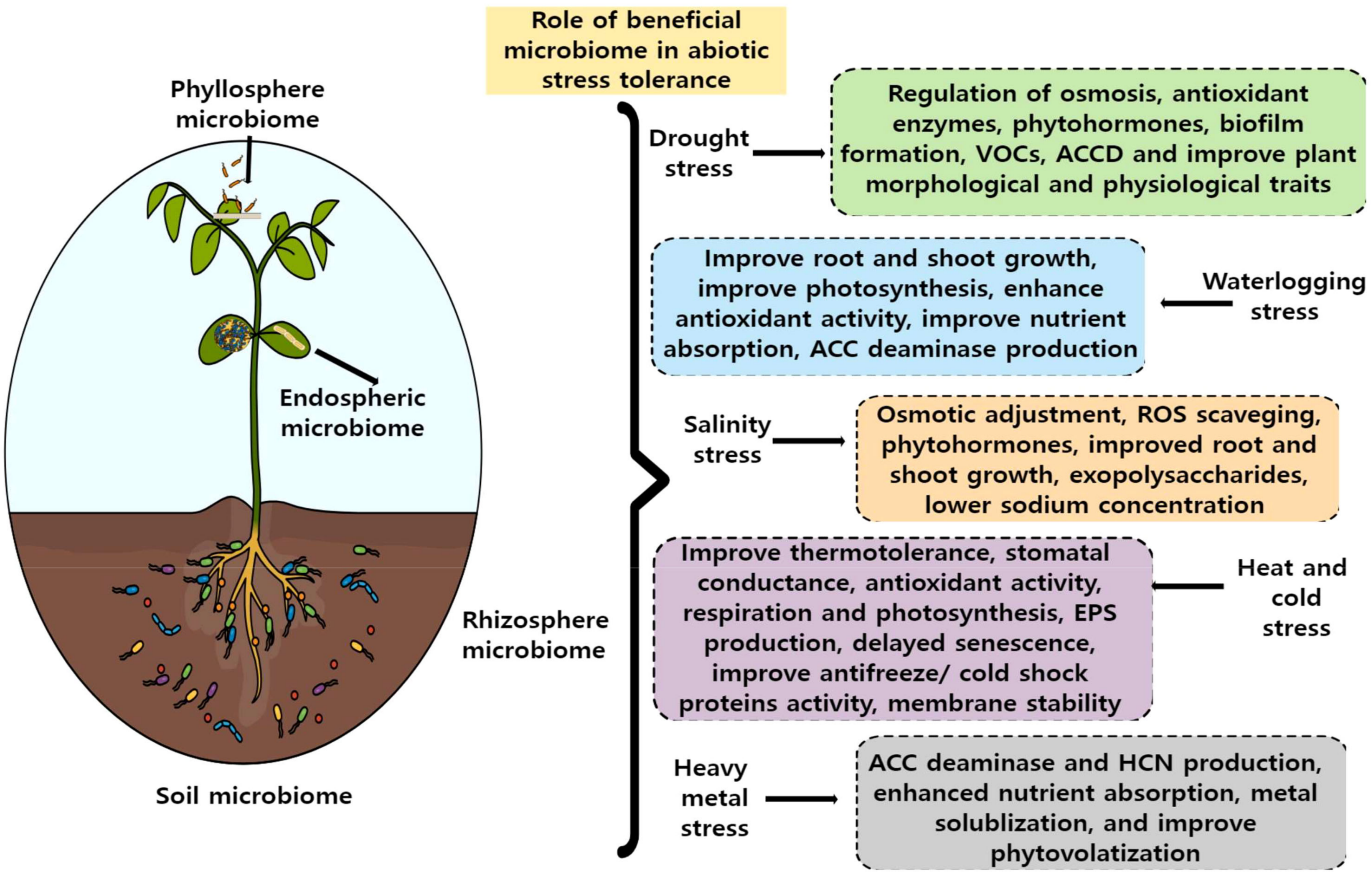
Role of plant beneficial microbiota in plant abiotic stress tolerance. Plant-beneficial microbial communities improve various morphological, physiological, biochemical, and nutritional traits in plants during abiotic stress conditions like drought, salt, heat, waterlogging, cold, and heavy metal stress. Some of the key functions that microbes involve to mitigate abiotic stressors are highlighted in the figure.

In nature, plants are associated with dynamic microbial communities of structurally and taxonomically diverse that offer a variety of beneficial services to them in terms of growth and adaptive response. For instance, beneficial microbes provide nutrients and modulate growth hormones by secreting auxins, gibberellins, and cytokinins. They also activate different defense hormonal pathways such as salicylic acid, jasmonic acid, ethylene, and abscisic acid which are the key regulators of biotic and abiotic adaptive responses (Ali et al., 2023). In order to combat abiotic stressors in plants, beneficial microbes perform a variety of functions, including activating the antioxidant system, osmobalancing, stomatal conductance, improving photosynthesis, water retention, improving root and shoot growth, and maintaining membrane stability, all of which are essential for plants’ survival in stressful situations **Figure 1**. Interestingly, some of the well-known microbial-assisted functions in plants are nitrogen fixation, phosphate solubilization, potassium solubilization, zinc solubilization, and iron sequestering. These processes not only benefit plant growth but also increase soil fertility and soil biota (Kamran et al., 2017; Mahmud et al., 2020). For instance, *Rhizobium, Azotobacter, Azospirillum, Gluconaceotobacter*, and *Burkholderia* are the most widely used nitrogen-fixing bacteria in sustainable agriculture (Aloo et al., 2022). The most important function of plant growth-promoting bacteria is secreting 1-aminocyclopropane-1-carboxylate (ACC) deaminase which breaks down ACC, an ethylene (ET) precursor and reduces the impacts of abiotic stresses such as salinity, drought, and waterlogging (Danish and Zafar-ul-Hye, 2019; Liu et al., 2019). Despite numerous reports of microorganisms having a variety of advantageous impacts we still know very little about the underlying molecular mechanisms. Customized microbial consortia can be developed for agricultural solutions if we have a mechanistic understanding of the interactions between plants and microbiota in heterogeneous abiotic stress environments. In nature, plants and their microbiome can very often face different combinations of multiple stressors such as drought and heat, heavy metal and cold, mechanical and other abiotic stresses, or a combination of biotic and abiotic stressors. It will be interesting to find out how these factors affect plant microbiome interactions and how will it affect the overall plant traits. We are still in the early stage of how combined stress affects plant microbiomes as most of the studies have focused on individual stress.

## Plant-microbe interactions during abiotic stressors

Environmental stressors not only affect plant growth and adaptive traits but also have a huge impact on plant microbiota and their interactions. Therefore, plants, microbes, and environmental stressors are highly interconnected hence their interactions are dynamic and complex. Understanding the effects of environmental stressors (biotic and abiotic) stress on plants, microbes, and their interactions is essential for developing more robust microbial consortia in sustainable agriculture. Also, the identification of beneficial microbes and antagonistic microbes during multiple stressors in plants will provide new frontiers for crop improvement (Busby et al., 2017). Abiotic stressors are known to affect plant microbiome structure and assembly as well as plant exudate composition. Root exudates are a crucial factors that influence the microbiome of plants when they are under abiotic stress. Root exudation plays a role in improving plant resource utilization and promoting communication between soil bacteria and plants in order to reduce stress. Plant root exudates consists both primary (sugars, amino acids, carboxylates) and secondary metabolites (coumarin sorgoleone, flavonoids) (Ali et al., 2022). Abiotic stresses have been demonstrated to trigger key root exudation components like abscisic acid, proline, trehalose, betaine, and pinitol (ABA), which either directly increase plant tolerance or indirectly do so by shaping beneficial microbiota (Gargallo-Garriga et al., 2018; Chai and Schachtman, 2022). Soil microbes, which frequently have a carbon shortage under stress conditions can obtain a significant energy source from exudates. As a result, a process known as “soil priming” occurs, in which the microbial community becomes more active and may release nutrients crucial for plants due to its function in nutrient cycle (Yan et al., 2023). Previous studies have shown that plants exposed to drought display major shifts in their root microbiome. For example, enrichment of actinobacteria (*Streptomyces* spps) was found in different plants after drought stress. These findings suggest that *Streptomyces* spp. may be actively recruited by plants and have favorable effects on them (Fitzpatrick et al. (2018). However, it remains unknown at the molecular level how plants shape *Actinobacteria* during drought stress. Recently, it was discovered that rehydrating sorghum plants following drought stress results in bacterial community rearrangement that resembles that of control plants (Xu et al., 2018). In contrast, after rewatering in rice plants there was no restructuring of microbial communities when compared to non-stressed plants. Hence, investigating how much the difference between sorghum and rice’s capacities to reconstruct the microbiota is a result of the host’s genetics, the local soil, or microbial variation would be intriguing. There are numerous studies that have utilized different microbes for mitigating drought stress. For instance, inoculation of wheat plants by *Burkholderia phytofirmans* (Naveed et al., 2014)., *Bacillus safensis* and *Ochrobactrum pseudogregnonense* (Chakraborty et al., 2013) enhance drought tolerance by improving photosynthetic activity, soil moisture, and nutrient and water absorption, antioxidant activity. On the other hand inoculation of *Azospirillum brasilense* in Arabidopsis increased drought resilience enhancing ABA levels which is hallmark of drough signaling in plants Cohen et al. (2015). Plants also enriched particular microbiota during salinity stress to mitigate salt stress. For example, in the rhizosphere of groundnuts, salt stress increased the relative abundance of cyanobacteria and *Acidobacteria* while decreasing the number of *Actinobacteria* and C*hloroflexi* (Xu et al., 2020). According to Yaish et al., 2016, salt stress induces significant changes in microbiome structure with more abundance of *Enterobacter* spp. A previous study has revealed that salt stress changes microbial communities in both below and above-ground plant parts (Hou et al., 2021b). Many salt stress-related hormones such ABA and salicylic acid (SA) have been found to drive the microbial community changes during salt stress in *Arabidopsis.* These studies provide evidence that plants recruit special beneficial microbiota that promotes growth traits under salinity. However, more studies are required in different crop systems using both salt tolerant and sensitive to further unravel how plants recruit specific microbial communities under salt stress. Temperature (heat and cold stress) also triggers microbial community changes in plants (Wipf et al., 2021; Liu et al., 2022). These studies have highlighted that plants recruit specific microbial taxa or genre during abiotic stressors which might help them to survive under such conditions. However, these studies were carried out under single stress. Therefore, future studies are required to examine how plants shape unique microbial communities during multiple stress combinations in both model and crop systems which can be further explored for mitigating combined stressors (drought +heat, drought +salinity, heat +salt stress etc) in sustainable agriculture. As a result, it is necessary to use new methods and technologies like machine learning that can be used to underpin how plants modify their microbiomes under multiple stress conditions and how stress microbiomes aid plant survival in such circumstances. Also, the identification of beneficial microbes and antagonistic microbes during multiple stressors in plants will provide new frontiers in microbiome ecology. Given that plant responses to microorganisms are typically highly species-specific, one crucial question is how compatible the plant microbiota implicated in plant stress tolerance will be across plant species and conditions (Marasco et al., 2013). For instance, some plants are more dependent on beneficial fungi and some on free living microbes therefore it will be interesting to identify microbes that confer greater stress tolerance to a variety of plants rather than individual plants.

## Development of tailored microbiota for plant growth promotion and multiple stress tolerance

Although the green revolution has dramatically increased crop production using chemical fertilizers, pesticides, and genetically improved crop cultivars but this achievement has been marred by numerous unsustainable practices, such as soil pollution, development of resistant pathovars, environmental damage, etc. Considering the future food demand under changing environmental conditions there is a need for a second green revolution but it should be achieved in a sustainable way. One way is to harness plant-beneficial microbiome or their products which provide eco-friendly services in terms of plant growth promotion and stress resilience (Arif et al., 2020; Ali et al., 2022; Ali et al., 2023). Microbes are known to flourish in extreme environments like high salinity, drought, heavy metal, high temperature, etc., and develop resistance to a variety of environmental insults and improper soil conditions. Therefore, it is obvious that microbes can assist plants under unfavorable environmental conditions and can be a more sustainable way to boost crop production and soil health. However, it will be intriguing to investigate if microorganisms can resist numerous stressors when they occur simultaneously or whether they act differently in response to individual stress.

**Figure 2:**
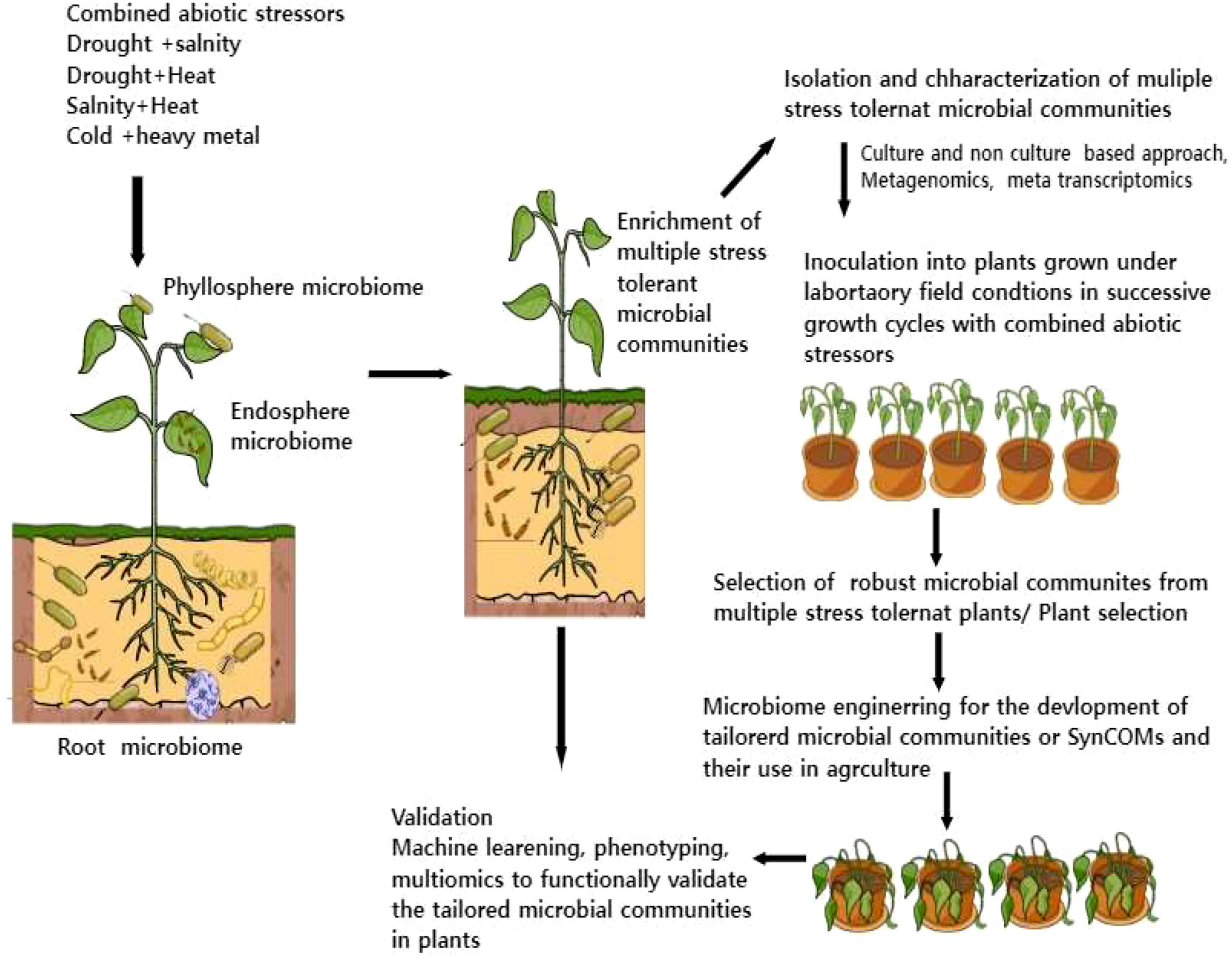
A systematic approach for the development of multiple stress-tolerant microbial communities in sustainable agriculture. This illustration highlights how plants could enrich selective microbial communities during combined stressors which can be further used for the development of tailored microbiota or SynComs using microbiome engineering. The role of omics tools, plant phenotyping, and machine learning for functional validation of tailored microbiota is also shown in the figure.

For the development of tailored multiple tolerant microbiota, we need to first identify the core microbiome and its interactions with the host, microbes, and environments. These studies require the integration of cutting-edge technologies like metagenomics, metatranscriptomics, and temporal-spatial mapping of plant responses and plant-colonizing bacteria and machine learning (Doni et al., 2022). Further, the development of beneficial multiple stress-tolerant microbiota in sustainable agriculture necessitates new model systems and prioritizes agricultural microbiome research. For instance, 1) we have to develop plant microbiome systems in both model and crop systems where we can study different host-microbiome interaction traits under combined stress conditions. 2) Selection of core microbiome under combined stress conditions using both culture and non-culture-dependent methods. 3) An essential frontier of plant microbiome interactions under stress combinations will be evaluating how plants undergo transcriptional, metabolic, and posttranslational alterations associated with root exudate chemistry, a crucial driver for core microbiome construction 4) Plant-based microbiome engineering involves plant breeding and genetic engineering for developing tailored microbiome is another aspect of harnessing beneficial core microbiome 5) Finally, microbiome engineering and enivroomics will also play key role in developing SynComs for multiple stressors in sustainable agriculture **Figure 2**. Some studies have also reported metaorganism-based microbiome engineering which involves the co-engineering of both plants and their associated microbes for particular metabolic traits. To address the above points there is a need to integrate different molecular (multi-omics) and system biology tools along with plant phenotype screening that will provide novel insights into the complexity of plant microbiome and improve models for the predictive traits necessary for a successful SynCom design.

## Conclusion

Recent developments in biotechnological tools and culture-independent techniques have revealed that plants are profoundly colonized with diversified microbial communities which are essential to their survival under changing environmental conditions. Nevertheless, over the past few years, our understanding of how plants and microbes interact under abiotic stressors has significantly increased, we still lack a thorough mechanistic understanding of how the microbiome shifts occurs under combined or multiple stressors. Therefore, future studies are required to decipher how plants enrich their microbiome during combined stressors and how they vary under different stress combinations. Also, how plants modulate root exudates during combined stressors is another key frontier in plant microbiome research as they are the major drivers for microbiome assembly and hence warrant further attention. Future research is needed to understand how phytohormones like ABA, SA, JA, and ET affect microbiome structure and how they contribute to microbiome-mediated stress tolerance in plants under multiple stressors. In this regard, reductionist methods under controlled lab conditions or utilizing SynComs will provide more mechanistic and functional insights into how combined abiotic stressors affect plant microbiome structure and how microbes enhance a plant’s ability to withstand abiotic stressors. A combination of metagenomics, metatranscriptomics, and temporal-spatial mapping of plant responses and plant-colonizing bacteria as well as the use of machine learning will aid to underpin how plants modify their microbiomes under multiple stress conditions which will aid in identifying unique and robust microbiota. For long-term usage of beneficial microbiota, it is necessary to identify bonafide receptors, genes, or transcriptional factors in plants that regulate microbiome drivers (root exudates, hormones) under combined stresses which can be employed in plant breeding or plant genetic engineering to create a desired robust microbiome for improving multiple stress resilience in crops. Additionally, the use of traditional and biotechnological-based microbiome engineering could also play a key role in developing multiple stress-tolerant beneficial microbiota for sustainable agriculture.

## Author contributions

SA: Conceptualization, Formal Analysis, Software, Supervision, Validation, Writing – original draft, Writing – review & editing. AT: Conceptualization, Software, Visualization, Writing – original draft, Writing – review & editing. IR: Conceptualization, Supervision, Writing – original draft, Writing – review & editing. YA: Writing – original draft, Writing – review & editing. RM: Writing – original draft, Writing – review & editing. HM: Conceptualization, Supervision, Writing – original draft, Writing – review & editing.
